# Oat-based milk alternatives: the influence of physical and chemical properties on the sensory profile

**DOI:** 10.3389/fnut.2024.1345371

**Published:** 2024-02-05

**Authors:** Roisin McCarron, Lisa Methven, Stephanie Grahl, Ruan Elliott, Stella Lignou

**Affiliations:** ^1^Department of Food and Nutritional Sciences, Harry Nursten Building, University of Reading, Reading, United Kingdom; ^2^Arla Innovation Centre, Agro Food Park, Denmark; ^3^Department of Nutrition and Exercise Sciences, Faculty of Health and Medical Sciences, University of Surrey, Guildford, United Kingdom

**Keywords:** oat, milk alternatives, sensory, physico-chemical, avenanthramides, GC-MS, particle size, LC-MS

## Abstract

**Introduction:**

Oat-based milk alternatives (OMAs) have become increasingly popular, perhaps due to their low allergenicity and preferred sensory attributes when compared to other milk alternatives. They may also provide health benefits from unique compounds; avenanthramides, avenacosides, and the dietary fibre beta-glucan. This has led to a variety of commercial options becoming available. Being a fairly new product, in comparison to other plant-based milk alternatives (PBMAs), means little research has been undertaken on the sensory profile, and how it is influenced by the physical and chemical properties.

**Methods:**

This study investigated the sensory, physical and chemical profiles of current commercially available OMAs, that varied in fortification, use of stabilisers, and oat content. The volatile compounds and their respective aromas were analysed using solid phase microextraction followed by gas chromatography mass spectrometry (GC-MS) and gas chromatography—olfactometry (GC-O). Liquid chromatography mass spectrometry (LC-MS) was used for identification of avenanthramides and avenacosides. Particle size and polydispersity index (PDI) were analysed using a Mastersizer and Zetasizer, respectively, with colour analysis carried out using a colourimeter, and viscosity measurements using a rheometer. Descriptive sensory profiling was used to assess the impact on the sensory characteristics of the different samples and the sensory data acquired were correlated with the instrumental data.

**Results:**

Samples with smaller particle size appeared whiter–both instrumentally and perceptually. The only clear plastic packaged product differed substantially in volatile profile from all other products, with a higher abundance of many volatile compounds, and high overall perceived aroma. Avenanthramides and avenacosides were present in all samples, but differed significantly in abundance between them.

**Discussion:**

The results suggested smaller particle size leads to whiter colour, whilst differences in processing and packaging may contribute to significant differences in aroma. Astringency did not differ significantly between samples, suggesting that the variation in the concentrations of avenacosides and avenanthramides were below noticeable differences.

## 1 Introduction

Plant based milk alternatives (PBMAs) have substantially increased in popularity, now accounting for around 8% of total retail “milk” sales in the UK (1). A shift away from cow’s milk may be due allergies and intolerances (2), and concerns over climate change, land and water use (3). Oat milk alternatives (OMAs) have received particular interest due to unique potential health benefits (2), including the presence of beta glucans — a dietary fibre shown to be beneficial in preventing diabetes, as well as lowering total blood cholesterol, and reducing the risk of cardiovascular diseases (4, 5). An increase in nut allergies (6) and soy allergies (7) make oat a popular alternative to other PBMAs. Oats also contain unique compounds with antioxidant properties, including avenanthramides (8), a group of phenolic compounds unique to oats, which have been shown to have anti-inflammatory effects (9), and avenacosides, which exhibit antifungal properties (10).

However, OMAs may face nutritional, sensory, and physicochemical challenges. The qualities of oat milk have not yet been fully investigated in comparison to other PBMAs such as soya, which has been on the market for over 70 years and for which there is extensive available research and literature (7). Despite being designed to have similar sensory properties to cow’s milk, some consumers find the difference in attributes to be unacceptable (11) whilst the nutritional value tends to be low in comparison to bovine milk (12).

The full production process of oats to OMAs is described by Zhang et al. (13) to include the following steps: dehulling groats, flaking, wet milling, enzyme hydrolyses, decanting, followed by additional ingredient formulation, ultra-heat treatment (UHT), and finally storage. However, with a variety of commercial OMAs available, it is possible these production steps may vary considerably, leading to varying sensory, and physicochemical properties. Processing can lead to a loss of vitamins and minerals (14), with a shorter UHT holding time enabling beverages to retain a higher level of vitamins during storage (13). Processing methods may also modify the physicochemical characteristics of beta glucans, with these effects being highly process-dependant and difficult to predict (15). Some differences may be due to drivers in industry, with some producers driven more by flavour cues focussing on how the product looks, acts and tastes, some influenced by concern over environmental or health claims, and others by nutrition (1).

The appearance of OMAs has been shown to have a significantly lower whiteness index than cow’s milk, making them easily distinguishable (7). A slight brownish colour may be due to natural pigments, and colour differences could result from differences in size and concentration of particles (16). Colour analyses on milks has shown those with lower particle size have a higher lightness index than those with larger particle size, as a result of light scattering (17). This may lead to an “off-white” colour some consumers could find unappealing (16). Bovine milk and soy milk contain only a small fraction of large particles, whilst oat milk has been shown to have twice as many large particles (>3 μm) as small particles (<3 μm) (18). Stability may affected by the polydisperse distribution of particles in OMAs, leading to an increased separation rate, with high amounts of sedimentation and creaming (7). Particle size has also been found to be highly correlated with stability in milks — with more stable milks measuring smaller particles (18). The high concentration of starch in OMAs can lead to increased viscosity (2), whilst the fortification of PBMAs may lead to chemical instability of the nutrients and nutraceuticals added (11).

Off-flavours in OMAs may result from the presence of unsaturated fatty acids and lipoxygenases that can lead to the formation of n-hexanal and n-hexanol, which are associated with a “beany” or “off” flavour (2). Production and storage can lead to lipid degradation and oxidation, causing the development of these off-notes (13). This may be problematic, as hexanal is considered a rancidity marker and may affect the acceptability of OMAs (19). In order to provide sensory attributes similar to those of cow’s milk, many OMAs contain stabilisers, emulsifiers, and flavourings (1).

The phenolic compounds in oats, including the previously mentioned avenanthramides, may also affect the sensory properties, as these have been found to correlate with bitter and astringent sensations (20). Astringent compounds can react with salivary proteins, leading to a loss of lubricity and result in a rough-tactile feeling in the mouth (21). However, it still remains to be established as to whether the phenolic compounds in OMAs are present in sufficient quantities to be perceived as astringent. Research has also shown that these phytate and oxalates present in oats may have anti-nutritional effects, reducing absorption of minerals (22). Cow’s milk naturally provides a variety of minerals including calcium, as well as vitamins B_2_, B_12_, A, and E, therefore OMA’s may be fortified to closer match this (11). However, mineral fortification may be undermined if absorption of the added minerals is hindered by phenolics, phytate and oxalates. Cow’s milk also contains proteins with all of the essential amino acids required by humans, being highly digestible and bioavailable (23), whereas OMAs are generally low in protein in comparison (12), with just 0.4–1% protein content (24). The protein in oats is also limited in the amino acid lysine, and may have poor digestibility (25).

Differences in the physicochemical, and volatile profiles may affect the taste, appearance, mouthfeel, and functionality of products. The sensory profile may be affected by astringency, resulting from avenanthramides and avenacosides, whilst differences in processing and packaging may possibly lead to off-notes. It is also possible that particle size and polydispersity index may lead to differences in stability and appearance. These differences found in individual products may affect overall acceptability of OMAs. Therefore, this paper focuses on the sensory, physicochemical and volatile profile of existing OMAs, and explores how these relate to one another. The aim is to identify specific compounds and properties in OMA’s and investigate how they contribute to the sensory profile. With such information it is anticipated that future developments in formulation and process optimisation may lead to an improved sensory profile of OMAs and increase consumer acceptability.

## 2 Materials and methods

### 2.1 Materials

#### 2.1.1 Oat milk alternatives

Six different OMA products were used for analyses, labelled; A, B, C, D, E, and F. These were standard commercial products from the UK market and selected based on the commercial availability, with factors such as price, accessibility and popularity taken into consideration, in order to accurately reflect a range of standard products for the industry. The selected samples had varying levels of fortification (Table 1) in order to analyse the potential effect of this fortification on resulting sensory attributes. The nutritional composition of the samples also varied, as shown in Table 2. All products selected were original versions—avoiding “barista,” flavoured, or sugar-free alternatives, to ensure comparable products were assessed. All samples were UHT long shelf-life products packed in paperboard carton packaging, aside from sample C which was packaged in clear plastic, stored refrigerated and had a shorter shelf life. During this study commercial production of product C ceased, however, analysis of this product was completed as the substantial differences between this and other products were of interest. Samples from the same batch code were used for each sample for the sensory and flavour analyses, with the samples opened and analysed within the same day for the sensory panel and GC-MS analyses. Mastersizer, Zetasizer and colourimeter analyses were also carried out within 24 h of opening from the same batch codes. Samples were then frozen, to be thawed at a later date for GC-O, LC-MS, and rheological analyses. Each analytical method was carried out in replicates from 3 separate cartons to account for batch to batch variation.

**TABLE 1:** Stated ingredients for the six products used in this study collected from online sources and/or product packaging at time of purchase.

Sample	Ingredients	Packaging	Shelf life
A	Oat base (water, oats 10%), rapeseed oil, calcium carbonate, calcium phosphates, salt, vitamins (D2, riboflavin, B12), potassium iodide	Liquid carton packaging (paperboard)	Long life
B	Oats (15%), water, rapeseed oil, salt	Liquid carton packaging (paperboard)	Long life
C	Oats 9%, oat flour 1%, plant fibre from citrus, water, salt	Clear plastic	Short
D	Oat base [water, oat (9.8%)], chicory root fibre, sunflower oil, calcium (tri-calcium phosphate), sea salt, stabiliser (gellan gum), vitamins (B2, B12, D2)	Liquid carton packaging (paperboard)	Long life
E	Water, oats (10%), rapeseed oil, tricalcium phosphate, calcium carbonate, salt	Liquid carton packaging (paperboard)	Long life
F	Spring water, organic gluten-free oats (11%), organic cold-pressed sunflower oil, sea salt	Liquid carton packaging (paperboard)	Long life

**TABLE 2:** Nutritional information of samples as stated on the product packaging at time of purchase.

Typical values	A	B	C	D	E	F
Energy (Kcal)	57	51	50	43	48	43
Fat (g)	2.8	1.5	0.8	1.5	2.1	1.6
Saturates (g)	0.3	0.3	0	0.1	0.2	0.4
Carbohydrate (g)	6.6	7.7	11	6.6	9.5	6.1
Sugars (g)	4.1	3.2	4.8	3.2	4.5	4.1
Fibre (g)	0.8		0	1.4		0.3
Protein (g)	1	1.3	0	0.3	0.2	0.8
Salt (g)	0.1	0.18	0.05	0.09	0.1	0.1
Vitamin B2 (mg)	0.21			0.21		
Vitamin B12 (μg)	0.38			0.38		
Vitamin D (μg)	1.1			0.75		
Potassium (mg)	151					
Calcium (mg)	120			120	120	
Iodine (μg)	22.5					

#### 2.1.2 Chemicals

For solid-phase microextraction (SPME), compounds used as standards were obtained from Sigma-Aldrich Co. Ltd. (Gillingham, UK): 1,2-dichlorobenzene (10 ppm in methanol) and the alkane standards C_6_–C_25_ (100 μg/mL) in diethyl ester. Sodium chloride, and HPLC grade water, methanol and hexane, were obtained from Fisher Scientific UK. LC-MS grade formic acid (98–100%) and acetonitrile were purchased from Merck (Darmstadt, Germany). Standards avenanthramide A (i.e., 2p), avenanthramide B (i.e., 2f), and avenanthramide C (i.e., 2c), avenanthramide D phyproof^®^, and avenacoside A (>95%), were purchased from Sigma Aldrich Co. Ltd. (Gillingham, UK).

### 2.2 Sensory analysis

For the sensory analyses, descriptive sensory profiling was carried out over the course of 2 weeks, using the trained sensory panel at the Sensory Science Centre (University of Reading) comprising of eleven panellists. All panellists had a minimum of 6 months experience, as well as four specific sessions training (30-min each session) on the OMA’s used during this study During week one, vocabulary development and training sessions contributed to the selection of thirty-five different attributes for scoring. To develop these attributes, coded samples were given to the panellists, and they were asked to describe appearance, aroma, taste, flavour, mouthfeel, and aftertaste/after-effects and produce as many descriptive terms as seemed appropriate. Reference materials (Supplementary Table 1) were used for assessors to confirm if the attribute was the appropriate descriptor. The vocabulary development and training sessions were carried out in a discussion room, whilst the quantitative sensory assessment took place in isolated sensory booths, each equipped with an iPad.

Once the consensus vocabulary was set, the panellists re-evaluated the OMAs and decided on anchors for the line scales. This led to an agreed profile of 8 appearance terms, 4 odour terms, 10 taste/flavour terms, 4 mouthfeel terms and 9 aftertaste/after-effects terms. Compusense Cloud Software (Compusense, Guelph, ON, Canada) was used to acquire the sensory data. The samples were provided in glass cups, with a saucer placed over the top—prepared approximately 5 min in advance of each sampling to create a headspace for aroma detection, tested at room temperature. The samples were randomly assigned two three-digit codes each (one for each of the two repeats) and given to the panel in a sequential balanced order. Over 3 days, the panel analysed each of the samples twice, and scored for each attribute using unstructured line scales (0–100). Panellists were instructed to sniff the samples first to score the aroma attributes, then assess the appearance before tasting (and swallowing) the samples to score the overall taste/flavour and mouthfeel attributes. There was a 30-s pause after the end of mouthfeel attributes and the panellists then scored the after-effects. Between samples, panellists cleansed their palate with water and crackers, with a 30-s pause between samples.

### 2.3 Instrumental analysis

#### 2.3.1 Volatile compounds

##### 2.3.1.1 Solid-phase microextraction followed by gas chromatography-mass spectrometry (SPME GC-MS)

Three millilitres of each sample were weighed into a SPME vial of 15 mL fitted with a screw cap and 0.5 g of sodium chloride was added along with 5 μL of 1,2-dichlorobenzene (10 ppm in methanol) as an internal standard. After equilibration at 40°C for 10 min, a 50/30 μm DVB/CAR/PDMS fibre was exposed to the headspace above the sample for 30 min. Four replicates from different product cartons were carried out over 3 days, in a randomised order each time. A blank run, using an empty carton, was used to ensure any volatile compounds from the lab or equipment were subtracted, as well as gain an indication of what compounds were present from the packaging. For this, a carton from Sample A was rinsed thoroughly and shaken with water, with 3 mL of this water analysed as a blank run. After extraction, the SPME fibre was inserted into the injection port of an Agilent 7890A-5975C gas chromatography mass spectrometer equipped with an automated injection system (CTC-CombiPAL). For the chromatographic separation, a capillary column HP-5MS (30 m × 0.25 mm × 0.25 μm film thickness) (Agilent, Santa Clara, CA, USA) was used. The oven temperature programme used was 2 min at 40°C isothermal and an increase 4°C/min to 250°C. Helium was used at 3 mL/min as carrier gas. The sample injection mode was splitless. Mass spectra were measured in electron ionisation mode with an ionisation energy of 70 eV, the scan range from 20 to 280 m/z and the scan rate of 5.3 scans/s. The data were recorded by HP G1034 Chemstation system. Volatile compounds were identified or tentatively identified by comparison of each mass spectrum with spectra from authentic compounds analysed in our laboratory, or from the NIST mass spectral database (26), or spectra published elsewhere. A spectral quality value of >80 was used alongside linear retention index (LRI) to support the identification of compounds where no authentic standards were available. LRI was calculated for each volatile compound using the retention times of a homologous series of C_6_–C_25_ *n*-alkanes and by comparing the LRI with those of authentic compounds analysed under similar conditions. The approximate quantification of volatile compounds was calculated from GC peak areas, by comparison with the peak area of the 1,2-dichlorobenzene standard, using a response factor of 1.

##### 2.3.1.2 Solid-phase microextraction followed by gas chromatography-olfactometry (SPME GC-O)

After extraction (using the same optimal extraction conditions as used for GC-MS), the SPME fibre was inserted into the injection port of an Agilent 7890B series ODO 2 (SGE) GC-O system equipped with an HP-5MS column (30 m × 0.25 mm × 0.25 μm film thickness). The outlet was split between a flame ionisation detector and a sniffing port. The injector and detector temperatures were maintained at 280 and 250°C, respectively. The oven temperature programme used was 2 min at 40°C isothermal and an increase 4°C/min to 250°C. Helium was used at 2 mL/min as carrier gas. Three assessors with normal olfactory function, from the Department of Food and Nutritional Sciences, were trained and carried out the procedure. Each assessor evaluated by sniffing each sample in duplicate and documented the odour description, retention time, and odour intensity (OI) on a seven-point scale (2–8), where <3 = weak, 5 = medium, and >7 = strong. *n*-Alkanes C_6_–C_25_ were analysed under the same conditions to obtain LRI values for comparison with the GC-MS data.

#### 2.3.2 Avenanthramides and avenacosides

##### 2.3.2.1 Sample preparation

The extraction was conducted according to Günther-Jordanland et al. (20), with some modifications to adapt from oats to oat-based milk. Each sample (10 mL) was placed into a separating funnel and 10 mL of hexane was added, shaken for 5 s and left to equilibrate for 15 min before removing the fat. The samples were then centrifuged at 4°C for 10 min at 9000 rpm, then the remaining hexane and fat layer was removed using a glass mini pipette. After this step, 500 μL of the sample was added to 1.5 mL of acetonitrile containing 50 μL of formic acid. This was then shaken for 1 h, centrifuged for 10 min at 9000 rpm, filtered using a 1.4 μm filter and analysed by LC-MS/MS. Each sample was analysed in triplicate.

##### 2.3.2.2 LC-MS/MS analysis

An aliquot (1μL) of the prepared sample was injected into a UPLC-MS/MS QQQ system, LCMS 8050 (Shimadzu) combined with Luna Phenyl-Hexyl (150 mm × 2.0 mm inner diameter, 5 μm, Phenomenex, Aschaffenburg, Germany) equipped with a guard column of the same type. Eluent A was composed of 0.1% formic acid in water, and Eluent B was composed of 0.1% formic acid in acetonitrile. Using a flow rate of 300 μL/min, the system was operated at 25°C, starting with 32% B under isocratic conditions for 1 min, then increasing the content of B to 70% over 3 min, followed by an increase to 100% B over 2 min, and keeping isocratic conditions for 3 min. Eluent was pumped down again to 32% over 2 min and held isocratically for a further 3 min. Analysis was performed in ESI- mode using the following MRM transitions: avenanthramide A: 298 > 254.15 298 > 133.9 298 > 159.85, avenanthramide B: 327.8 > 284.25, 327.8 > 268.1, 327.8 > 160.85, avenanthramide C: 314 > 178.2, 314 > 134.85, 314 > 134.2, avenanthramide D: 282 > 238.2, 282 > 118.95, 282 > 144.85, avenacoside A: 1061.7 > 899.3, 1061.7 > 163. Dwell time was 10 ms for each transition and Q1, collision energy and Q3 voltages were optimised using standards of each compound.

The calibration curves were run with a linear curve fit, a weighting of 1/C^2, and were not forced through the origin. A quantitative method with external standards was used; avenanthramide A, B, C (referred to in some literature as Bp, Bf, and Bc, respectively) and D, as well as avenacoside A. According to literature avenanthramides A, B and C are the three major forms in oats (9), with avenacoside A as another primary component (27), and thus a targetted approach was followed searching for these compounds. Each standard was diluted with 75% acetonitrile, 25% water, in order to match the sample conditions for solvent composition. Quantifier ions used for identification were; avenanthramide A 298 > 254, avenanthramide B 328 > 284, avenanthramide C 314 > 178, avenanthramide D 282 > 238, and avenacoside A 1107.05 > 1061.45, respectively. Data acquisition and quantification was performed in Labsolutions Insight software (Shimadzu).

#### 2.3.3 Colour analysis

Using a colourimeter, Konica Minolta Chroma metre CR-400, CIELAB system (illuminant C, 10° viewing angle, with an 8 mm diameter port), three repeated measurements were obtained for each sample. The samples were held in a glass cell (diameter 60 mm × 15 mm) and the lightness (L*), red/green coordinate (± a*) and yellow/blue coordinate (± b*) were recorded to give a measure of the lightness and colour.

#### 2.3.4 Particle size analysis

A Malvern Mastersizer S was used to obtain measurements of particle size [suitable for readings above 1 μm (1000 nm)]. Three repeats were carried out, one after the other on the instrument, with the water flushed out between each reading to reduce residual particles. A Malvern nanoseries ZS zetasizer was used to obtain measurements of the polydispersity index. Polydispersity index is a measure of the heterogeneity of a sample based on size, and is determined by dynamic light scattering (28). Each sample (1 ml aliquot in a cuvette) was measured in triplicate, with three technical replicates per aliquot. Data were recorded and analysed using the Malvern zetasizer software. Default settings were selected with Angle 173, with run conditions 25°C for 200 s.

#### 2.3.5 Rheological properties

Rheological properties were studied using a controlled stress rheometer (MCR 302, Anton Paar Ltd. St Albans, UK) using parallel plate geometry (50 mm diameter). OMA samples were frozen and thawed prior to rheological analyses. The gap size was 1 mm and a resting time of 300 s prior to measurement was established for sample relaxation and temperature equilibration. Apparent viscosity was measured as a function of shear rate over the 1 to 1000 s^–1^ range, at 25°C. Measurements were carried out in triplicate for each of the samples, with an average viscosity calculated for each at shear rate 50 s^–1^.

### 2.4 Statistical analysis

The quantitative data for each compound identified in the GC-MS and LC-MS analyses, or physicochemical measurements (colour, particle size, PDI, viscosity) were analysed by one-way analysis of variance (ANOVA) using XLSTAT Sensory (Version 2022.5. 1. 1388). For those compounds or physicochemical parameters exhibiting significant difference in the one-way ANOVA, Tukey’s honest significant difference (HSD) test was applied for multiple pairwise comparisons. SENPAQ (Qi Statistics, Kent, UK) was used to carry out ANOVA and principal component analysis (PCA) using the covariance matrix, of the sensory panel data. For the sensory data two-way ANOVA was used where the samples were fitted as fixed effects and the assessor as random effects, and both of these treatments were tested against the sample by assessor interaction. Tukey’s HSD *post-hoc* test was applied for pairwise comparisons. In all multiple pairwise comparisons, significance was assumed at *p* ≤ 0.05. Multiple factor analysis was applied to correlate the means for the sensory data (taken over the assessors) with the means of volatile data.

## 3 Results

### 3.1 Sensory analysis

The trained sensory panel agreed to use 35 terms for the quantitative assessment of the samples and the mean panel scores for these attributes are shown in Table 3. Overall, 16 out of 34 attributes were significantly different between the six samples. The panellists’ individual results were analysed for repeatability and reliability. No obvious anomalies were observed as the panel scored to a consistent standard with one another.

**TABLE 3:** Mean panel scores for sensory attributes of the six OMA samples.

Attributes	Mean score (0–100)^a^						Significance of sample (*p*-value)^b^
	**A**	**B**	**C**	**D**	**E**	**F**	
**Aroma**							
Overall intensity	32.8^a^^b^	26.9^b^	41.4^a^	33.3^a^^b^	33.5^a^^b^	35.8^a^^b^	0.002
Sweet	18.0	16.3	22.6	20.9	19.6	21.0	0.172
Wet oat	25.4^a^^b^	18.8^b^	33.6^a^	27.3^a^^b^	27.1^a^^b^	31.1^a^	0.002
Malt	12.7^a^	2.7^b^	10.0^a^^b^	8.3^a^^b^	7.9^a^^b^	6.6^a^^b^	0.031
Nutty	3.7	1.8	5.9	5.9	4.3	7.8	0.196
Stale	7.0	8.0	8.4	4.4	8.9	6.2	0.670
Single cream	0.4	1.7	0.4	3.0	0.5	2.8	0.183
Brown bread	12.9^a^^b^	1.6^c^	16.8^a^	10.8^abc^	15.5^a^^b^	6.4^bc^	0.0001
**Appearance**							
Off white colour	56.4^a^^b^	52.3^b^	67.3^a^	50.5^b^	39.3^c^	31.0^c^	<0.0001
Glass cling	41.8^a^	29.9^b^	24.8^b^	28.9^b^	26.0^b^	35.6^a^^b^	<0.001
Froth/foam	47.2^a^	43.6^a^	26.3^b^	37.7^a^^b^	39.9^a^^b^	46.9^a^	<0.001
Bubble size	25.9^a^	26.3^a^	14.5^b^	24.5^a^	26.6^a^	28.2^a^	<0.0001
**Taste**							
Sweet	27.1^a^^b^	25.4^a^^b^	21.9^b^	30.4^a^	28.7^a^^b^	25.1^a^^b^	0.034
Bitter	12.2	12.3	15.0	9.2	9.7	14.5	0.087
Acid/tang	3.6	7.5	9.8	4.6	5.5	7.3	0.162
Metallic	8.0	9.6	9.4	7.1	7.6	8.8	0.801
**Flavour**							
Malty	9.7	4.9	6.8	10.5	6.6	4.8	0.260
Wet oats	29.3	27.9	32.6	29.0	29.8	33.1	0.434
Nutty	6.0	6.9	6.2	9.0	9.2	12.2	0.105
Stale	6.7	5.3	7.2	3.8	5.7	4.4	0.653
Single cream	7.3^a^^b^	9.5^a^	0.0^b^	9.2^a^^b^	10.4^a^	9.2^a^^b^	0.024
Brown bread	11.5^a^^b^	4.9^b^	14.8^a^	10.1^a^^b^	11.5^a^^b^	6.2^a^^b^	0.008
**Mouthfeel**							
Mouthcoating	32.7	32.0	23.3	26.7	27.3	29.7	0.101
Body	31.5^a^	26.4^a^	18.2^b^	28.8^a^	26.5^a^	30.3^a^	<0.001
Powdery	13.8^a^^b^	22.8^a^	22.4^a^	7.0^b^	7.1^b^	6.7^b^	0.0001
Astringency	14.3	18.8	21.4	13.8	17.6	16.9	0.135
**Aftertaste**							
Bitter	10.6	9.2	14.0	9.0	9.2	12.6	0.288
Metallic	8.4	10.5	11.5	6.0	7.5	8.8	0.068
Sweet	16.5^a^^b^	20.2^a^	13.3^b^	17.5^a^^b^	19.6^a^^b^	16.5^a^^b^	0.040
Wet oats	19.4	19.6	23.4	19.8	21.2	24.1	0.387
Single cream	3.1	8.3	0.4	8.0	7.9	5.3	0.023
**After effects**							
Mouthcoating	18.4	18.6	13.0	17.9	16.6	14.9	0.225
Powdery	11.1	12.9	13.6	4.8	5.6	4.8	0.006
Astringent	19.8	18.3	18.8	14.4	16.7	16.5	0.467
Salivating	20.6	23.5	23.5	20.7	22.2	24.4	0.700

*^a^*Means not labelled with the same letters are significantly different (p < 0.05); means are from two replicate samples.

*^b^*Probability of a significant difference between samples.

Significant differences were found in all appearance attributes, with sample C displaying the most off-white colour, yet the least froth/foam, bubble size and glass cling. Samples E and F were found to have significantly less off-white colour than all other samples, whilst sample A displayed the most glass-cling. For aroma, significance was found within the overall aroma intensity, wet oats, malt and brown bread aromas. Overall intensity was highest in sample C, as well as wet oats and brown bread, yet the malty aroma was highest in sample A. In terms of significant differences in taste, sample D was found to be the sweetest, with sample C the least sweet. For flavour, sample C was the highest in the brown bread note, yet was the only sample to score no single cream flavour at all. Significant differences were found in the mouthfeel of the samples, with A scoring the highest in body, whilst sample C was the lowest. Samples B and C also had significantly more powdery mouthfeel than all other samples. For aftertaste, B was found to be the most sweet, with sample C again being the least. There were no significant differences for any other after effects. Although there were no significant differences between samples for astringency, within mouthfeel or as an after-effect, astringency was perceived in all samples. The relationship between this astringency and non-volatile compounds was further evaluated.

### 3.2 Instrumental analysis

#### 3.2.1 Volatile compounds

##### 3.2.1.1 GC-MS–Optimisation of the extraction conditions

In order to determine the optimal conditions for volatile extraction, sample A was used with varying conditions, based on previous studies of other plant-based alternatives (29, 30), as well as bovine milk (31). The following parameters were evaluated: incubation and extraction temperature (40°C and 50°C), incubation time (10, 20 and 30 min), extraction time (10, 20, and 30 min), and salt (sodium chloride) addition varied from 0, 0.5, 0.75, and 1 g. Optimal conditions were selected considering the overall amount of the extracted volatiles. Increasing salt from 0 to 0.5 g resulted in an improved efficacy of the extraction, however, increasing above 0.5 g showed no additional effect, therefore 0.5 g was selected. Additionally, increasing incubation time above 10 min showed no obvious differences, whereas increasing the extraction time from 10 to 30 min resulted in more abundant peaks. Finally, increasing the temperature above 40°C did not improve efficacy of the procedure, therefore 40°C was selected as the incubation and extraction temperature. In conclusion, the optimal parameters were set at 40°C, 10 min incubation time, 30 min extraction time, and addition of 0.5 g of NaCl.

##### 3.2.1.2 SPME GC-MS

More than 35 compounds were identified in the headspace of the six samples (Table 4) including four esters, eleven aldehydes, five ketones, four terpenes, one alkane, three alkenes, three alcohols, and four furans.

**TABLE 4:** Volatile compounds identified in the headspace of six samples analysed by SPME GC-MS.

Compounds	LRI^a^	Aroma descriptor^b^	Estimated quantities^c^						Significance (*p*-value)^d^
			**A**	**B**	**C**	**D**	**E**	**F**	
**Esters**									
Methyl acetate	515	Sweet	1.57^bc^	1.17^c^	3.73^a^	2.17^abc^	3.56^a^^b^	1.08^c^	0.001
Methyl propanoate	629	Fruity, rum	8.49^a^	6.28^a^^b^	2.62^b^	6.36^a^^b^	8.55^a^	7.06^a^	0.003
Methyl butanoate	720	Fruity, creamy	11.37^a^	7.74^a^^b^	4.76^b^	9.52^a^^b^	12.82^a^	9.46^a^^b^	0.013
Methyl 2-methylbutanoate	775	Fruity	5.46^a^	5.56^a^	2.12^b^	4.57^a^^b^	5.21^a^^b^	3.60^a^^b^	0.020
**Aldehydes**									
2-Methylpropanal	552	Wet cereal, straw	1.21^bc^	0.70^c^	2.48^a^^b^	1.24^bc^	2.87a^a^	1.37^abc^	0.002
3-Methylbutanal	649	Fruity	6.29^b^	2.35^b^	15.10^a^	7.08^b^	14.40^a^	2.34^b^	<0.0001
2-Methylbutanal	659	Cocoa	5.11^bcd^	2.39^cd^	10.06^a^^b^	6.10^bc^	7.77^a^^b^	1.93^d^	<0.0001
Hexanal	802	Green	94.29^c^	31.76^d^	207.09^b^	54.71^cd^	40.86^cd^	316.31^a^	<0.0001
2-Hexenal	853	Green	nd^c^	nd^c^	1.13^b^	nd^c^	nd^c^	1.89^a^	<0.0001
Furfural	836	Bready	nd^a^	nd^a^	2.65^a^	11.96^a^	nd^a^	nd^a^	0.163
Heptanal	903	Green	3.05^cd^	1.59^d^	6.39^a^	5.75^a^^b^	4.17^bc^	6.65^a^	<0.0001
(2E)-Heptenal	951	Green	nd^b^	nd^b^	2.37^b^	23.52^a^	5.92b	4.92^b^	<0.0001
Benzaldehyde	959	Almond	1.38^b^	1.26^b^	2.12^a^	0.92^bc^	0.47^c^	1.34^b^	0.000
Octanal	1007	Fruit-like	nd^b^	nd^b^	2.97^a^	nd^b^	2.61^a^	nd^b^	<0.0001
Non-anal	1087	Rose-orange	nd^b^	nd^b^	5.10^a^	nd^b^	nd^b^	nd^b^	0.00
**Ketones**									
Butanedione	593	Buttery	2.33^c^	1.84^c^	3.58^abc^	5.35^a^^b^	6.04^a^	2.51^bc^	0.001
2-Butanone	598	Sharp sweet	34.38^a^^b^	20.47^bc^	12.69^c^	21.60^bc^	42.90^a^	25.24^abc^	0.002
2-Methyl-3-pentanone	749	Mint	nd^c^	6.87^a^	3.21^c^	nd^c^	nd^c^	nd^c^	<0.0001
3 Methyl 2-butanone	661	Camphor	11.10^a^	10.08^a^	3.76^b^	8.73^a^^b^	11.72^a^	9.10^a^^b^	0.007
6-Methyl-5-hepten 2-one	787	Citrus, fruity	nd^b^	nd^b^	5.16^a^	nd^b^	nd^b^	nd^b^	<0.0001
**Furans**									
2-Methylfuran	603	Chocolate	0.67^bc^	0.16^c^	1.26^b^	0.94^bc^	2.37^a^	1.00^bc^	<0.0001
3-Methylfuran	611		0.40^a^	nd^b^	0.32^a^	nd^b^	0.36^a^	0.27^a^^b^	0.000
2-Ethylfuran	700	Malty, beany	4.53^b^	0.57^d^	2.66^cd^	0.45^d^	1.74^cd^	6.18^a^	<0.0001
2-Pentyl furan	992	Fruity, green	15.50^b^	2.46^c^	12.62^b^	4.55^c^	3.88^c^	33.23^a^	<0.0001
**Alkanes**									
Octane	800	Gasoline	12.66^c^	132.70^a^	6.32^c^	49.52^b^	120.59^a^	32.43^bc^	<0.0001
1-Octene	794	Gasoline	nd^c^	nd^c^	0.76^c^	14.10^b^	18.65^a^	nd^c^	<0.0001
(E)-2-Octene	804		nd^c^	1.25^c^	0.91^c^	61.49^a^	33.34^b^	nd^c^	<0.0001
(Z)-2-Octene	811		nd^b^	1.17^b^	1.08^b^	31.06^a^	30.96^a^	nd^b^	<0.0001
**Terpenes**									
α-Pinene	739	Pine	1.88^b^	186.64^a^	1.78^b^	0.34^b^	nd^b^	9.78^b^	0.000
β-Pinene	978	Woody green, pine	nd^b^	35.71^a^	nd^b^	nd^b^	nd^b^	0.51^b^	<0.0001
Limonene	1034	Citrus	0.60^b^	5.63^b^	40.08^a^	0.71^b^	0.59^b^	nd^b^	0.037
Camphene	951	Woody	nd^b^	16.411^a^	nd^b^	nd^b^	nd^b^	nd^b^	<0.0001
**Alcohols**									
Pentanol	763	fermented	9.77^bc^	2.62^cd^	12.15^d^	4.76^cd^	2.38^d^	27.94^a^	<0.0001
Hexanol	867	Herbal	4.08^b^	nd^b^	0.98^b^	nd^b^	nd^b^	55.01^a^	<0.0001
Octen-3-ol	969	Mushroom	0.88^a^	2.72^abc^	4.30^c^	3.46^bc^	2.29^a^^b^	4.09^bc^	<0.0001

*^a^*Linear retention index on a HS-5MS column.

*^b^*Aromas obtained from TheGoodScents company, and PubChem.

*^c^*Estimated quantities (ng) collected from the headspace of 3 mL of OMA sample calculated by comparison with 20 μl of 10 ppm 1,4-dichlorobenzene used as internal standard; means (from three replicate samples) not labelled with the same letter in a row were significantly different (p < 0.05); as determined by Tukey’s Honestly significant difference (at *p* = 0.05); nd, not detected.

*^d^*Significance of sample effect (*p*-value).

Of the esters, sample C was significantly lower in methyl propanoate, methyl butanoate, and methyl 2-methylbutanoate, however, it exhibited the highest abundance in methyl acetate. Samples A and E were generally higher in esters, both being the highest in methyl butanoate and methyl propanoate, whilst A, E and B were significantly higher than C in butanoic acid.

Hexanal was the most abundant aldehyde in the samples, and was significantly higher in sample F, followed by sample C. Sample D exhibited significantly more 2-heptenal and furfural than all others, whilst sample C was found to have high abundance in octanal, 3-methylbutanal and 2-methylbutanal, and was the only sample to contain non-anal. Sample E was the second highest in 3-methylbutanal, 2-methylbutanal and octanal, whilst being the highest in 2-methylpropanal. Sample B was generally found to have low levels of aldehydes such as 2-methylpropanal and heptanal, with no 2-hexanal, furfural, 2-heptenal, octanal or non-anal being detected. B was also significantly lower than all others aside from F in 3-methylbutanal and 2-methylbutanal. Despite being lower in 3-methylbutanal and 2-methylbutanal, F exhibited the highest abundance in heptanal and 2-hexenal, a compound only present in C and F.

Of the alcohols detected, sample F was significantly higher than all others in hexanol and pentanol. Octen-3-ol was present in all samples, with C exhibiting significantly higher abundance than A and E.

In terms of ketones, sample E was abundant in 2-butanone, butanedione, and 3 methyl 2-butanone. 6-methyl-5-hepten-2-one was only detected in sample C, whilst 3-methyl-2-pentanone was detected in only B and C.

Of the furans, 2-pentylfuran was significantly higher in sample E, followed by, A, and then C. Sample B exhibited the lowest abundance in all furans aside from 2-ethylfuran, which it was found to be the second lowest, following sample D.

Most terpenes, including alpha-pinene, beta-pinene and camphene were highest in sample B, whereas limonene was the highest in sample C.

**FIGURE 1:**
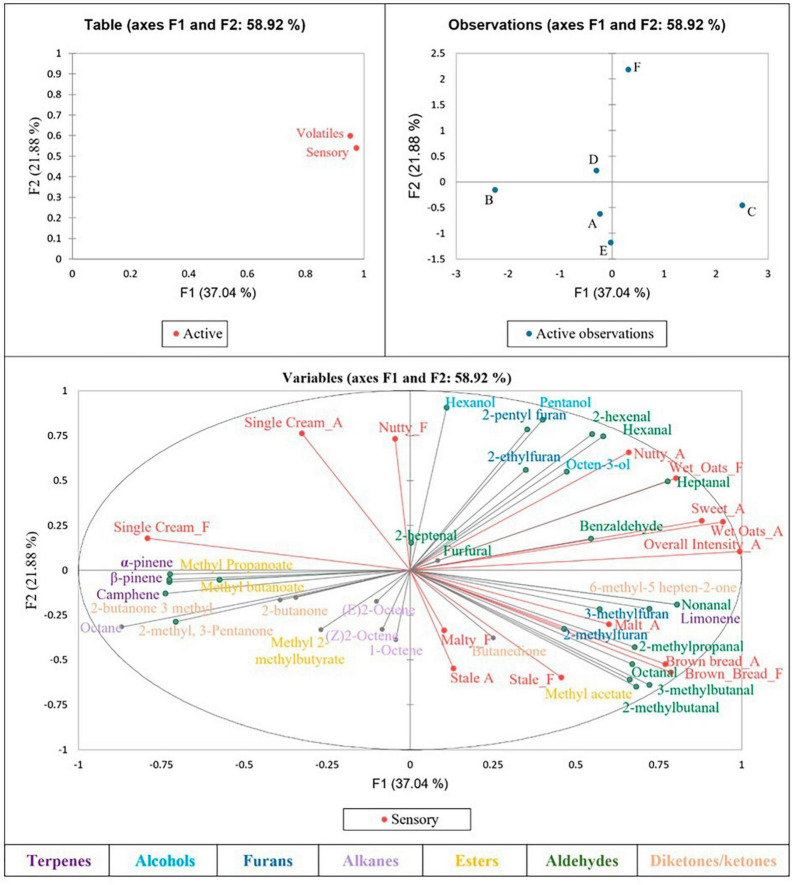
Multiple factor analysis correlating sensory data with volatile results.

Multiple factor analysis was used to determine correlations between the sensory results and volatile compounds for each sample (Figure 1), from which multiple significant correlations were found. Brown bread aroma was found to be significantly positively correlated with 2-methylpropanal, 3-methylbutanal and 2-methylbutanal—all branched chain aldehydes described as having a malty and chocolate aroma (32), as well as with methyl acetate. Brown bread flavour was also significantly positively correlated with methyl acetate, 2-methylbutanal and 3-methylbutanal. 3-methylbutanal is an amino acid-derived key flavour compound in bread, with a fairly low taste threshold (32), which may have resulted in the brown bread aroma and flavour correlation.

A nutty aroma was found to be positively correlated with heptanal, a compound typically described as having a fatty aroma when in isolation (33). Sweet and wet oats aroma were also both found to be significantly positively correlated with heptanal, yet negatively correlated with methyl 2-methylbutanoate.

Wet oats flavour was shown to be positively correlated with hexanal, which often imparts a green aroma (33), as well as with pentanol, 2-hexenal and heptanal, yet was again negatively correlated with methyl 2-methylbutanoate. Single cream flavour was significantly positively correlated with methyl propanoate and 3-methyl-2-butanone, whilst being negatively correlated with benzaldehyde, 6-methyl-5-hepten-2-one, limonene and non-anal.

Sensory attributes malty, stale and single cream aroma, and malty, nutty and stale flavour, were not found to be significantly positively or negatively correlated with any compounds identified in the samples.

##### 3.2.1.3 SPME GC-O

Gas chromatography-olfactometry analysis of the samples yielded a total of 24 distinct odorants in the chromatogram that were of note due to multiple panellists perceiving them, which are presented in Table 5. General aroma intensity of all samples was fairly low with only a few strong odours.

**TABLE 5:** Mean GC-O scores from 3 assessors.

Odour description^a^	LRI^b^	Compound	Confidence^c^	A	B	C	D	E	F
Milk/butter/cheese	<600	Unknown				2			
Butter	<600	2-methylpropanal	A			3			4
Caramel/buttery	<600	Butanedione	A	3.5	5.3	5	3.5	3.8	3.3
Cocoa	651	2-methylbutanal	A			4			
Sulphur/toast	667	Unknown			4.3		3	2	
Boiled milk	675	Unknown		2		2			3
Oat milk/buttery	741	Unknown				2		2	3
Fruity/sweet	767	Methyl 2-methylbutanoate	A			3.5		2	
Green/citrus	801	Hexanal	A	3.4	4.4	4	2.7	3	3.7
Soily/herb	849	Unknown					2.5	5	
Marmite/yeast	873	2-methyl-3-furanthiol	B		4.5		3.5	3.3	4
Soily/wood	885	Unknown		2.7					
Potatoes	874	Unknown		3.3	5		4.7		
Soup/bread/potato	911	Methional	B	4.3	4.8	3.8	3.7	3.5	3.6
Coffee	913	2-furanmethanethiol	B		6				5
Starch/wheat/wet bread	918	Unknown			3.5				5
Cereal/buttery biscuits	930	2-acetylpyrroline	B		3	4		3	3
Mushroom	982	Octen-3-ol	A	4.2	3.8	4.6	5.2	4.2	5.2
Green chemical	992	Unknown			4	3	3.5	3.3	
Soil/mushroom/mould/coffee/wood	1097	2-isopropyl-3-methoxypyrazine	B	2	4.5	5.3	3	3.3	
Toasted bread/smokey meat	1099	Guaiacol	B	4	4.3	4.3	3		3
Ink/chemical	1104	2,6-dimethylphenol	B						4
Sweet milk/caramel	1116	Unknown			3.7				
Cocoa/makeup powder/dusty/soil	1161	Unknown		3.5	4	4.3	2.5		4

*^a^*Odour description given by assessors (some terms were grouped together due to similarity of the meaning).

*^b^*Linear retention index calculated from a linear equation between each pair of straight chain n-alkanes C_6_–C_25_.

*^c^*Confidence in accuracy of associated compounds; A = LRI in agreement with those of authentic compound–compound present in the GC-MS results; B = LRI in agreement with those of authentic compounds, however, the compound was not present in the GC-MS results. The associations with the compounds found, were based on aroma descriptions from Pubchem, and thegoodscentscompany, and the LRIs and quantities found from an internal database with compounds analysed under similar conditions in our lab.

Six aromas detected by GC-O were identified in the GC-MS analysis. From Table 5, it can be seen that multiple compounds present in the GC-MS results can be directly associated with detected aromas, due to similar descriptors and LRIs. These included 2-methylbutanal, methyl 2-methylbutanoate, hexanal, and octen-3-ol. Aromas such as buttery and caramel/buttery with LRIs of 552 and 593 (both below 600) in the GC-O were identified as 2-methylpropanal and butanedione based on the GC-MS results.

Other aromas are likely to have resulted from volatile compounds that were not identified by the GC-MS, however, their descriptors and LRIs closely match specific odour compounds present in an internal database used in our lab with authentic compounds ran and analysed at similar conditions. These included 2-methyl-3-furanthiol, methional, 2-furanmethanethiol, 2-acetylpyrroline, 2-isopropyl-3-methoxypyrazine, guaiacol, and 2,6-dimethylphenol. Eleven other aromas were found within the GC-O results, however, an associated volatile compound for these aromas was not found, suggesting they were highly odour active compounds and below the limit of detection of the GC-MS.

#### 3.2.2 Avenanthramides and avenacosides (LC-MS/MS)

**FIGURE 2:**
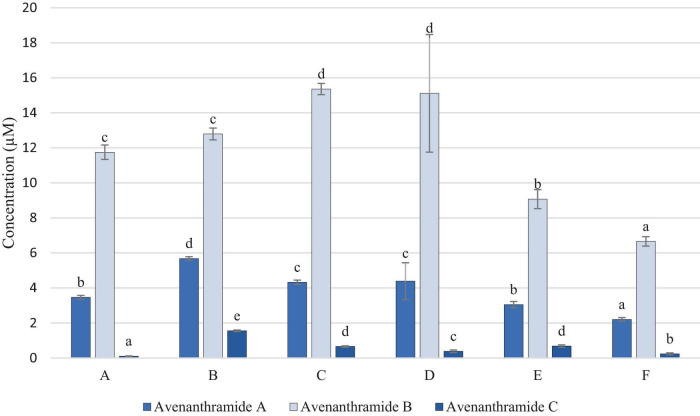
Concentration of Avenanthramides (μM in OMA sample). Data represents means of three instrumental replicates of three sample replicates ± standard deviations (*p*-value < 0.0001). Differing small letter represent sample significance from multiple comparisons as determined by Tukey’s honestly significant difference (at *p* = 0.05).

Figure 2 shows that avenanthramides A, B and C were present in all samples. Avenanthramide D was measured above the limit of detection (LOD), however, was below the limit of quantification (LOQ) in all samples, and therefore has not been included in the results.

Avenanthramide B was detected in the higher concentrations in all samples, in comparison to avenanthramides A and C, which is to be expected due to it being the most abundant of these compounds in oats. Figure 2 shows that samples C and D were significantly higher in avenanthramide B, than all other samples, whilst sample F was significantly lowest in both. However, avenanthramides A and C, were found to be significantly highest in sample B.

**FIGURE 3:**
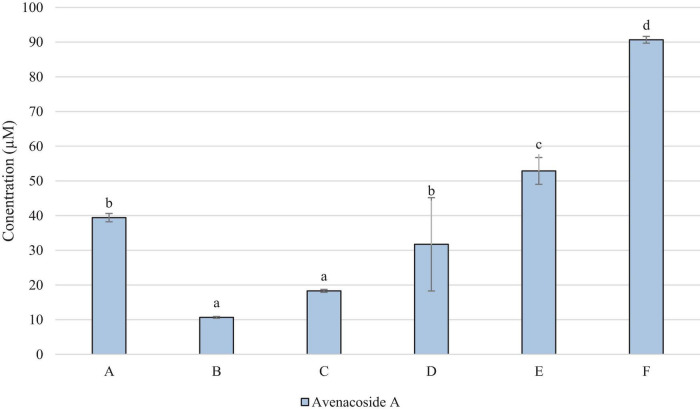
Concentration of Avenacoside A (μM in OMA sample). Data represents means of three instrumental replicates of three sample replicates ± standard deviations (*p*-value < 0.0001). Differing small letter represent sample significance from multiple comparisons as determined by Tukey’s Honest significance difference (at *p* = 0.05).

Figure 3 demonstrates that avenacosides were present in higher concentrations than avenanthramides in the OMA samples. However, the levels did not follow the same patterns with avenanthramides, with sample F being significantly highest, and B and C significantly lower in avenacoside A than all other samples.

#### 3.2.3 Colour analysis

**FIGURE 4:**
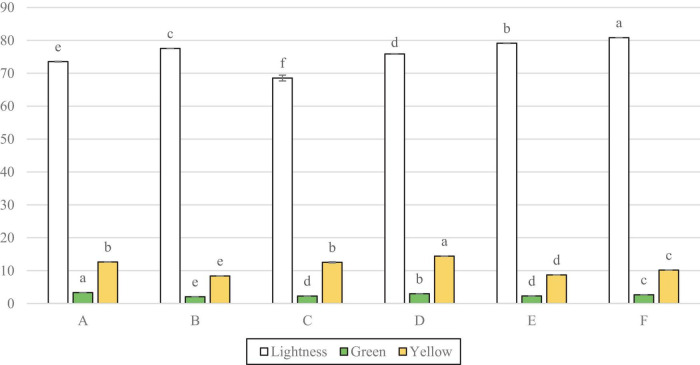
Colorimetre readings for lightness (L*) green direction (a*) and yellow direction (b*). Data represents means of three replicates ± standard deviations (*p*-value < 0.0001). Differing small letter represent sample significance from multiple comparisons as determined by Tukey’s HSD (at *p* = 0.05). Green note was measured in minus values, converted to positives for clarity on graph.

Figure 4 shows that the lightness for all samples was significantly different. Sample C measured the lowest lightness and F the highest, followed by E. However, figure 4 also shows that D, F, and A all had significantly more green note than C, whilst D had significantly more of a yellow colour.

#### 3.2.4 Particle size analysis

**FIGURE 5:**
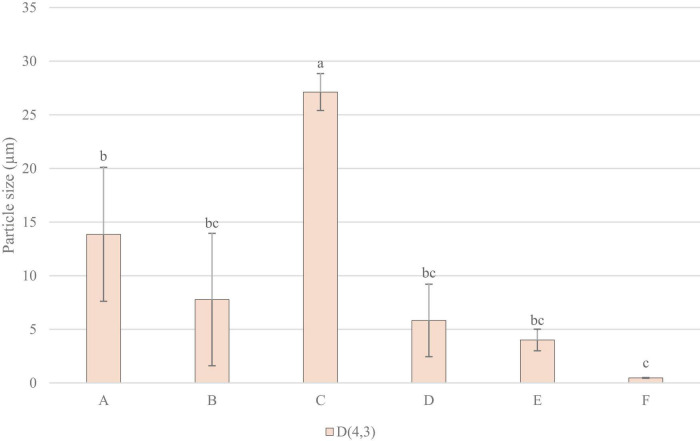
Particle Size Measurements: D, 4, 3 (volume weighted mean/mass moment mean diameter) mm. Data represents means of three replicates ± standard deviations (*p*-value < 0.0001). Differing small letter represent sample significance from multiple comparisons as determined by Tukey’s HSD (at *p* = 0.05).

Figure 5 shows that sample C had significantly larger particle size, being the highest in volume weighted mean, surface weighted mean, and median particle size. Sample E and F generally measured lower in particle size, with F measuring the lowest in volume weighted mean, and E the lowest in surface weighted mean, and median particle size.

**FIGURE 6:**
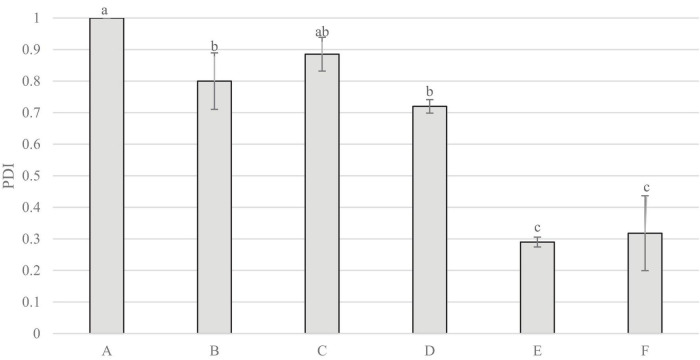
Polydispersity Index (PDI) of non-filtered samples. Data represents means of three replicates ± standard deviations (*p*-value < 0.0001). Differing small letter represent sample significance from multiple comparisons as determined by Tukey’s HSD (at *p* = 0.05).

Figure 6 indicates that sample A measured the highest reading for polydispersity index, significantly higher than all others, aside from C. Samples E and F measured significantly lower polydispersity index than all other samples.

#### 3.2.5 Rheological properties

**FIGURE 7:**
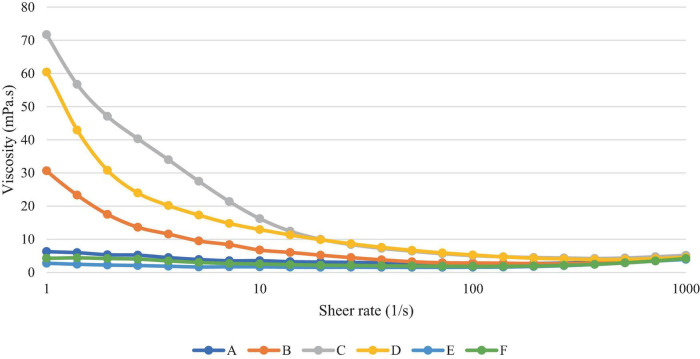
Sample viscosity as a function of shear rate.

Figure 7 shows that all samples decreased in viscosity with increasing shear rate, indicating that these products show non-Newtonian shear thinning behaviour (*N* < 1). Samples B, C and D were higher in viscosity and a larger drop in viscosity with increasing shear rate, in comparison to samples A, E and F, at lower shear rates (<100^–1^). At higher shear rates, however (>500^–1^), shear thickening behaviour can be observed, with a slight increase in viscosity in all samples. However, it may be the case that the viscosity recordings above 500^–1^ are outside of the experimental limit, leading to this increase in viscosity as a result of experimental error (34). Sample C contained the highest carbohydrate content as shown in Table 2 and was also found to have the highest viscosity at lower shear rates.

A comparison of samples at shear rate 50 s^–1^ is often used as this is thought to represent the shear rate of the oral cavity, however, it is important to note that there more recent studies have shown a large range of sheer rates from 1 to 1000 s ^–1^ (35). The mean values of viscosity of the samples at this shear rate are given in Table 6. Samples C and D were found to demonstrate a significantly higher viscosity than all others at this shear rate, whilst sample E was significantly lower, at 1.54 mPa.s.

**TABLE 6:** Viscosity values of samples at shear rate 51.8 (1/s).

Sample	A	B	C	D	E	F
Viscosity (mPa.s)	2.83^bc^	3.24^c^	6.42^d^	6.68^d^	1.54^a^	1.95^ab^

Data represents means of three replicates. Differing small letter represent sample significance from multiple comparisons as determined by Tukey’s HSD (at *p* < 0.05).

#### 3.2.6 Extent of separation

**FIGURE 8:**
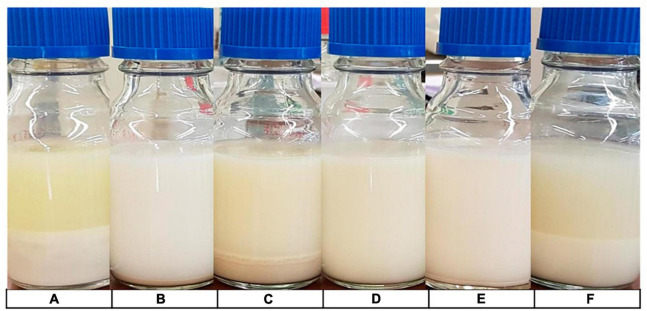
Extent of separation. Samples stored in glass vials for 24 h at 5°C for visual comparison of separation. Order from left to right: **(A–F)**.

Figure 8 demonstrates the visible separation after 24 h in samples A, C and F. With sample A having the highest PDI, and C the highest volume weighted mean, this suggests that increased PDI and particle size may relate to increased separation. However, it does appear from figure 8 that separation is also occurring in sample F, despite having a much lower particle size and PDI. It is not entirely clear as to why this may be the case, however, it is likely that this is due to other factors such as stabilisers used; Table 1 shows that F did not contain any stabilisers, whilst some of the less separated samples contained stabilisers including plant fibres and gellan gum.

## 4 Discussion

### 4.1 Avenanthramides and avenacosides in relation to ingredients and sensory attributes

Oat concentration did not appear to be directly influencing the perceived astringency of samples, as samples highest in oat content did not score higher in astringency. Nor was a trend found between protein content and astringency, despite an association within literature between protein and astringency (36). Avenanthramides and avenacosides are known to contribute to both astringency and bitter taste (20); sensations that were present at low levels in all samples. However, the samples did not differ in these attributes; samples with higher concentrations in these compounds were not detected as more astringent or bitter. Therefore, we suggest that the differences in avenanthramides and avenacosides between samples were below their just-noticeable-difference thresholds.

It is possible that the levels of astringency may be affected by other factors, including acids, dehydrating agents, and salts (37). In addition, the lipid content of the samples may have masked the perception of astringency (38). Sample C had the lowest fat content, and sample A had the highest (Table 2). However, although the mean astringency value was highest in C and amongst the lowest in A, which fits the lipid hypothesis, these differences in mean astringency values were not significant. Overall, whether the level of astringency and bitterness found in the samples would be detected by untrained consumers, and whether it is a factor in consumer acceptance, requires further investigation.

It is apparent from the LC-MS results that the levels of avenanthramides and avenacoside did not reflect the oat content (Table 1); with samples C and D containing the lowest quantities of oats, yet the highest overall concentration of avenanthramides. Sample B contained the highest quantity of oats, yet the lowest concentration of avenacoside A. With oats being the only possible source of these compounds, this may suggest that loss is occurring throughout stages of production. Phenolic compounds in oats have been found to decrease by 85% after the first 6 months of storage and remain at that level for the rest of the 12 months storage (39). This may suggest that the differences in phenolic compounds found within the 6 samples, may be related to storage time. Differences in the oat genotype and growing conditions may also affect the concentration of avenanthramides in the oat material (40). The differences in concentrations between avenanthramides and avenacosides, may suggest that certain compounds are more or less susceptible to degradation or loss than others. Steaming of oats has previously been shown to moderately reduce content of avenanthramide A, yet not affect avenanthramides B and C (9). Total avenanthramide concentration in oats has been found to range from 1.2 to 79.7 mg/kg, depending on the genotype (41). The total concentration of avenanthramides A, B, and C combined in the samples ranged from 8.6 to 25.9 μM, which is the equivalent of 0.00973–0.0293 mg/kg. This is substantially lower than what has been found in the literature, however, it is important to consider the high moisture content of the samples, in comparison to pure dehulled oat grain.

Within the sensory results sweet taste was found to be significant, with sample D measuring a significantly sweeter taste than sample C. This does not, however, match the nutritional information shown in Table 2, which shows D and B to contain the least quantity of sugar, with C the most. It is not clear as to why this may be the case other than these differences in sugars being below the threshold of noticeable differences for taste. The sweet taste perception may also be more complicated than just the reported level of sugar, due to the presence of complex carbohydrates (42).

These findings suggest that the effect of ingredients on the sensory profile is complicated and therefore may be difficult to predict.

### 4.2 Relationships between volatile compounds and sensory attributes

Steaming of oats has been shown to increase the concentration of certain compounds, including 3-methylbutanal, benzaldehyde, heptanal, hexanal, and 2-pentylfuran, whilst the combined effect of kilning and steaming may boost the amount of Maillard reaction-related volatiles (43). This may suggest that samples with higher concentrations of these compounds may have been affected by processing. Of these, a significantly higher abundance of benzaldehyde, was measured in sample C. Benzaldehyde has been suggested to likely result from interactions of reducing sugars and amino acids (44), and has been shown to significantly increase during storage of processed oats (39). Many other volatile compounds were also significantly higher in sample C, including nonanal, 6-methyl-5-hepten-2-one, limonene, and octen-3-ol. This increased abundance of many volatiles in sample C may have resulted in the significantly higher overall aroma as determined by the sensory panel, as well as the total of 15 distinct aromas detected in the GC-O analysis – the joint highest amount alongside sample B. Processed oats have been found to emit a higher odour than native oats (39), again suggesting this sample may have been affected by processing.

Differences in oat varieties have been shown to significantly affect sensory characteristics, including oat aroma, bitter taste, metallic, oat and creamy flavour, and oats and metallic after flavour (45). This may have contributed to the differences seen in the sensory results. Sample C was found to have the highest stale flavour by the sensory panel, although not significantly, as well as containing significantly higher 3-methylbutanal than all other samples. This may be associated with the reference of flaked almonds used by the panel for the attribute of stale, as 3-methylbutanal is described as one of the most predominant compounds in roasted almonds (46). This could also be related to the presence of benzaldehyde, an aroma associated with almonds (33), in which sample C measured significantly higher than all other samples. Methyl acetate, 3-methylbutanal, octanal, non-anal, 6-methyl-5 hepten-2-one and limonene, are all described as a sweet or citrus aromas, and could potentially have influenced the highest sweet aroma found in the sensory results, and the highest caramel note and fruity/sweet aromas within the GC-O results.

Hexanal, which has been shown to be the predominant aldehyde in oats (47), was also found to be the most abundant aldehyde in these samples. Hexanal is a lipid oxidisation product, which has been shown to increase during storage periods of processed oats (39), and therefore may have been affected by photo-oxidation from the clear plastic packaging in sample C, leading to the increased abundance. Photo-oxidation in oats may occur during processing, and is one of multiple reactions that can trigger the formation of lipid derived volatile compounds that may exhibit off-flavours (43). Differences in hexanal may also result from the possibility of a protein rich kernel to eliminate hexanal, as well as from variability of process conditions (48), having been shown to rapidly rise after heat treatment (47).

Sample B had the lowest mean overall aroma intensity, brown bread and wet oats aroma as determined by the sensory panel. This may have been influenced by the limited volatiles, as this sample was also found to have the lowest mean abundance in 16 of the compounds measured in the GC-MS; significantly lower than sample C in 14 compounds. Sample B exhibited the lowest abundance in all furans aside from 2-ethylfuran, in which it was found to be the second lowest, following sample D. Thermal processing is reported to be a main cause of furan formation, occurring to a large extent during the Maillard reaction (49).

Multiple factor analysis also found multiple correlations between the sensory results and volatile compounds, including sweet, wet oats and nutty aromas with heptanal, and brown bread aroma with methyl acetate, 2-methylpropanal, 3-methylbutanal and 2-methylbutanal. Wet oats flavour was shown to be positively correlated with pentanol, hexanal, 2-hexenal and heptanal. Single cream flavour with methyl propanoate, and 3 methyl 2-butanone, and brown bread flavour with methyl acetate, 3-methylbutanal and 2-methylbutanal. Such correlations do not necessarily indicate that the volatile compounds are responsible for the resulting flavours, they can be incidental correlations where volatiles group together. However, from the literature we can reflect that some volatile compounds may be influencing these sensory characteristics; for example branched chain aldehydes, specifically 3-methylbutanal, being flavour compounds associated with bread (32), whilst positively correlating with brown bread aroma and flavour.

The stale aroma which was detected in low levels in all samples by the sensory panel may be related to the highly scoring mushroom aroma picked up in every sample during the GC-O and identified as octen-3-ol. Despite the low concentrations of octen-3-ol detected, this compound has a very low odour threshold (>1 ppb) and may indicate an off-odour in oats (50). Octen-3-ol is produced by lipid oxidation, and increases greatly with storage time (51), therefore due to oats high lipid content, and active lipolytic enzymes (50), this may have resulted in the octen-3-ol detected within these samples. The sweet aroma detected in all samples may relate to the caramel note detected highly within all samples in the GC-O results and identified as butanedione.

### 4.3 Lightness, particle size, and rheology properties

The sensory results demonstrated that samples F and E were perceived to have the least off-white colour, with sample C the most. A similar finding was presented in the colourimeter results, as C was again found to have the least lightness, with F measuring the highest lightness, followed by E. The colour measurements did not find C to have more red or green colour than the other samples, which suggests that the off-white colour perceived by the panel, is due to lack of lightness, rather than influence from colour. The particle size measurements showed samples F and E to be the smallest, which may have influenced the increased lightness as a result of light scattering (17), and thus supports the hypothesis that lower particle size may contribute to increased whiteness.

The sensory results demonstrated that C was significantly more powdery than D, E, and F, as well as having the most powdery after effect. This also corresponds with particle size, as sample C was measured to have the largest volume weighted mean, and F the least, suggesting that larger particles may be resulting in a powdery mouthfeel and after-effect.

The rheology measurements found sample C to be the most sheer thinning during the lower sheer rates (below 10 mPa.s), followed by B and then D. This negatively correlates with the fat content listed in the nutritional information, with sample C containing the lowest fat content, followed by B and D. Sample C also exhibited the highest particle size which may have affected the rheology. OMAs in general have been shown to have a higher viscosity than other plant-based milks, potentially due to a higher carbohydrate content (52). This is also seen in sample C with the highest carbohydrate content, and generally higher viscosity at lower sheer rates.

This study may have been affected by slight limitations in analytical conditions. Due to the various methods used, it was not possible to carry out all of the sensory, chemical and physical analyses on the same batch at the same time. Also due to product C ceasing to be manufactured, which was out of the control of the study, all products needed to be frozen and thawed between sets of analyses. In ideal conditions all samples would have been analysed fresh, after opening the same day. However, all of the six products were analysed in the exact same conditions, all being frozen and thawed at the same time, therefore the difference in findings between the samples will still be an accurate representation.

## 5 Conclusion

The combined results from all six samples help to conclude influences between the physicochemical, sensory, and volatile properties, along with effects from ingredients and packaging. These include the likelihood that smaller particle size may lead to increased lightness and less perceived off-white colour, as well as reduced powdery mouthfeel—which may be beneficial for the sensory profile. Smaller particle size, as well as the potential addition of stabilisers may both contribute to a decreased separation. The results also suggested that certain compounds were detected in higher abundances in the GC-MS analysis in the clear-packaged non-UHT sample—potentially resulting from photo-oxidation. This appeared to have contributed to perceived aromas through GC-O, and through sensory results, suggesting that the avoidance of clear packaging may help to prevent off-notes.

The results also demonstrated that avenanthramides and avenacosides were present in all samples, which may be beneficial for determining the nutritional value of oat-based milk alternatives. These compounds were not only present, but significantly different between samples and not directly related to the differences in oat content. This may suggest that future analyses on the effects of processing, storage and packaging on these compounds, may be very beneficial to ensure preservation. The lack of significant differences between the samples for bitter taste and astringency, despite differences in avenanthramides and avenacosides, may suggest that there is the potential to increase these compounds without having a negative effect on the sensory profile. These results may be useful in combination for considerations of future OMA development, to improve the sensory profile and nutritional content going forward.

## Data availability statement

The raw data supporting the conclusions of this article will be made available by the authors, without undue reservation.

## Ethics statement

Ethical review and approval were not necessary for this study as the study involved tasting standard commercial samples by a trained sensory panel that are employees and have consented to taste and rate food as part of their job.

## Author contributions

RM: Data curation, Formal analysis, Investigation, Methodology, Resources, Software, Validation, Visualization, Writing – original draft, Writing – review and editing, Conceptualization. LM: Conceptualization, Funding acquisition, Methodology, Supervision, Writing – review and editing. SG: Supervision, Writing – review and editing. RE: Conceptualization, Funding acquisition, Supervision, Writing – review and editing. SL: Conceptualization, Funding acquisition, Methodology, Software, Supervision, Validation, Writing – review and editing.
