# Supplementary material for: Oat-based milk alternatives: the influence of physical and chemical properties on the sensory profile
Source: Front Nutr. 2024 Feb 5;11:1345371. doi: 10.3389/fnut.2024.1345371 (PMC10877596; doi:10.3389/fnut.2024.1345371)
Supplement: Supplementary file 1 [file Table_1.docx]

**Supplementary material**

**Table S1**: Summary of attributes with references and/or description used to confirm the developed vocabulary with the panel.

| Modality | Attribute^a^ | Reference and/or Descriptor^b^ |
| --- | --- | --- |
| Appearance | Off-white colour | D: amount of darkness or colour away from pure white |
|  | Froth/foam | D: visual foam on surface of sample without stirring |
|  | Bubble size | D: visual perceived size of bubbles on surface of sample without stirring |
|  | Glass cling | D: visual residue of sample on glass vial |
| Aroma | Overall intensity | D: strength of all aromas combined |
|  | Sweet | Ref: aqueous solution of sucrose |
|  | Nutty | Ref: blended mixed nuts |
|  | Wet oats | Ref: oats + cold water, soaked overnight |
|  | Single cream | Ref: single cream |
|  | Malt | Ref: barley malt extract |
|  | Stale | Ref: flaked ground almonds |
|  | Brown bread | Ref: sliced brown bread |
| Taste | Bitter | Ref: aqueous solution of quinine |
|  | Sweet | Ref: aqueous solution of sucrose |
|  | Acid | Ref: aqueous solution of citric acid |
|  | Metallic | Ref: iron sulphate 0.0036g/L |
| Flavour | Stale | Ref: flaked ground almonds |
|  | Brown bread | Ref: sliced brown bread |
|  | Nutty | Ref: blended mixed nuts |
|  | Wet oats | Ref: oats + cold water, soaked overnight |
|  | Single cream | Ref: single cream |
|  | Malt | Ref: barley malt extract |
| Mouthfeel | Mouthcoating | D: residue of sample in mouth |
|  | Powdery | D: grainy or large particle size |
|  | Astringency | Ref: tannic acid 0.2g/l |
|  | Body | D: sensation of palate fullness and viscosity |
| Aftertaste | Bitter | Ref: aqueous solution of quinine |
|  | Metallic | Ref: iron sulphate 0.0036g/L |
|  | Sweet | Ref: aqueous solution of sucrose |
|  | Single cream | Ref: single cream |
|  | Wet oats | Ref: oats + cold water, soaked overnight |
| After-effects | Mouth coating | D: residue of sample in mouth after swallowing |
|  | Powdery | D: grainy or large particles left in mouth after swallowing |
|  | Astringency | Ref: tannic acid 0.2g/l |
|  | Salivating | D: saliva produced after swallowing |

^a^ All anchors nil to extreme; ^b^ Ref = reference used, D = descriptor used to confirm attribute to panel.
